# De Novo Transcriptome Assembly and Annotation Elucidate the Response to Extreme Temperature Stress in the Intermediate Host *Bulinus globosus* of *Schistosoma haematobium*

**DOI:** 10.3390/ijms26115326

**Published:** 2025-06-01

**Authors:** Xinyao Wang, Jianfeng Zhang, Ying Yang, Suying Guo, Yinlong Li, Zhiqiang Qin, Hamza Juma, Saleh Juma, Kun Yang, Shizhu Li, Jing Xu

**Affiliations:** 1 National Key Laboratory of Intelligent Tracking and Forecasting for Infectious Diseases, National Institute of Parasitic Diseases at Chinese Center for Disease Control and Prevention (Chinese Center for Tropical Diseases Research), NHC Key Laboratory of Parasite and Vector Biology, WHO Collaborating Centre for Tropical Diseases, National Center for International Research on Tropical Diseases, Shanghai 200025, China; wangxinyao@jipd.com (X.W.); yangying@nipd.chinacdc.cn (Y.Y.); guosy@nipd.chinacdc.cn (S.G.); lily@nipd.chinacdc.cn (Y.L.); qinzq@nipd.chinacdc.cn (Z.Q.); lisz@chinacdc.cn (S.L.); 2 National Health Commission Key Laboratory of Parasitic Disease Control and Prevention, Jiangsu Provincial Key Laboratory on Parasite and Vector Control Technology, Jiangsu Provincial Medical Key Laboratory, Jiangsu Institute of Parasitic Diseases, Wuxi 214064, China; zhangjianfeng@jipd.com (J.Z.); yangkun@jipd.com (K.Y.); 3 Ministry of Health of Zanzibar, Zanzibar P.O. Box 236, Tanzania; jhamza977@gmail.com (H.J.); salehjuma2003@yahoo.com (S.J.); 4 School of Public Health, Nanjing Medical University, Nanjing 211166, China; 5 School of Global Health, Chinese Center for Tropical Diseases Research, Shanghai Jiao Tong University School of Medicine, Shanghai 200025, China

**Keywords:** *Bulinus globosus*, climate change, de novo transcriptome, *Schistosoma haematobium*, survival, extreme temperature stress

## Abstract

Schistosomiasis remains a major global public health challenge. *Bulinus* serves as an intermediate host for *Schistosoma*, including *S. haematobium*, *S. intercalatum*, and *S. guineensis*. Emerging evidence suggests that temperature fluctuations associated with global climate change are key factors influencing the survival and distribution of *Bulinus*. The ecological shifts in intermediate host snails may significantly influence schistosomiasis transmission dynamics, thereby exacerbating threats to human health. However, the physiological effects of temperature stress on the survival of *B. globosus* at the molecular level, including gene expression and underlying mechanisms, remain unclear. Our experimental study found that extreme temperature stress significantly reduced the survival rates of *Bulinus globosus* (*B. globosus*). De novo transcriptome sequencing revealed key genes associated with lipid metabolism, carbohydrate metabolism, homeostasis regulation, and the antioxidant system. Kyoto Encyclopedia of Genes and Genomes (KEGG) pathway analysis identified significant enrichment of differentially expressed genes (DEGs) in heat shock protein pathways, propanoate metabolism, and N-acylethanolamine metabolism pathways. Overall, this work provides the first transcriptomic characterization of the thermal stress response in *B. globosus*, extending genomic resources for annotation and stress-related gene discovery. These findings establish a solid foundation for developing control strategies to mitigate climate-driven risks of schistosomiasis transmission.

## 1. Introduction

Schistosomiasis remains a major global public health concern, prevalent primarily in more than 70 countries and regions across Asia, South America, the Middle East, and Africa [1]. Among the species of *Schistosoma* that infect humans, *Schistosoma japonicum* (*S. japonicum*), *S. haematobium*, and *S. mansoni* are the most common pathogenic species, while *S. intercalatum*, *S. mekongi*, and *S. guineensis* exhibit a more limited geographical distribution [2]. It is estimated that there are 230 million infections worldwide, with 85% concentrated in sub-Saharan Africa. Notably, over two-thirds of these infections are caused by *S. haematobium* [3,4]. Unlike other *schistosome* species that primarily cause intestinal symptoms, *S. haematobium* is associated with urogenital diseases and has well-documented causal links to bladder cancer [5,6]. Urogenital damage, particularly among women, has been well established as a significant factor contributing to the transmission of human immunodeficiency virus (HIV) [7,8].

*Bulinus* represents a genus of hermaphroditic freshwater snails that comprises 37 described species, some of which serve as intermediate hosts for various *Schistosoma* species, including *S. haematobium*, *S. intercalatum*, and *S. guineensis* [9]. These snails are widely distributed across the African continent, including Madagascar and smaller oceanic islands, as well as Mediterranean islands, southern continental Europe, and southwestern Asia [10]. Based on shell morphology and other organismal characteristics, four major groups of *Bulinus* species have been identified. This research conducts experimental work based on the China–Zanzibar–WHO cooperation project for schistosomiasis elimination. The goal of the government of Zanzibar is to eliminate schistosomiasis. The intermediate host of this disease is mainly *B. globosus*. This study aims to provide a theoretical basis for the elimination of schistosomiasis and for risk monitoring in Zanzibar and other countries [11]. *B. globosus*, recognized as the predominant intermediate host of *S. haematobium*, plays a crucial role in the transmission of schistosomiasis across Africa, especially in Zanzibar [10,12]. *B. globosus* is widely distributed across Africa, the islands of the Indian and Atlantic Oceans, the Mediterranean region, and adjacent areas in the Middle East. Previous studies have shown that the annual regeneration of *B. globosus* can be sustained by the current climate conditions in mainland China, with a northward expansion trend observed in a study conducted from 2015 to 2019 [13]. This indicates that *B. globosus* could spread and colonize countries or regions where environmental conditions are favorable.

*Bulinus* is a poikilothermic animal that adjusts its body temperature according to the surrounding environment. These animals require less energy to maintain their body temperature and physiological functions, allowing for a greater proportion of the energy obtained from food to be allocated to growth [14]. However, their adaptability to environmental changes is relatively limited [15]. Previous studies have identified temperature as the key factor influencing the survival and distribution of *Bulinus* spp. [16,17]. The findings by Wang et al. [13] revealed that the mortality rate in the low-temperature group decreased as the temperature increased, while the mortality rate in the high-temperature group conversely increased. The median lethal temperature (ET50) was determined through regression analysis. The minimum ET50 values for snail eggs, juveniles, and adults were 8.5, 7.0, and 7.0 °C, respectively, while the maximum ET50 values were 36.6, 40.5, and 40.2 °C. Extreme temperatures, both too low and too high, can lead to mortality.

*Bulinus* spp. can resist extreme temperatures through various physiological adaptations [18]. Numerous studies have explored the physiological responses of mollusks. For instance, the secretion of an epiphragm aids mollusks in reducing the risk of freezing and enhances their tolerance to cold conditions. Some researchers have examined the effects of extreme temperature stress on energy metabolites and specific respiratory enzymes in *Oncomelania*. Their findings indicate that energy substances, succinate dehydrogenase (SDH), and cytochrome oxidase (CCO) in the soft tissue of *Oncomelania* are influenced by temperature stress [17,19]. The tolerance of mollusks is closely linked to the metabolic functions of these organisms. However, the physiological effects of temperature stress on the survival of *B. globosus* at the molecular level, including gene expression and underlying mechanisms, remain unclear.

Transcriptome assembly refers to the reconstruction of RNA transcripts using RNA-seq data, aided by an existing reference genome for the organism. Despite the significant role of *Bulinus* in the elimination of *S. haematobium*, there is a notable lack of in-depth molecular-level studies conducted on these snails [20]. Genomic information essential for understanding the biology of basic organisms is scarce for the genus *B. globosus* [21], particularly for non-model organisms for which no genome data are available. In such cases, RNA transcripts are reconstructed de novo from raw RNA-seq reads without a reference genome [22]. In this study, *B. globosus* was selected as the research subject to identify genes associated with severe temperature stress through de novo transcriptome data. This approach provides insights into the molecular mechanism of *B. globosus* in response to temperature stress at the transcriptional level, rebuilding on the *Bulinus* RNA-seq dataset, and offers valuable genomic resources for *Bulinus* genomics, such as genome annotation and gene mining. This study aims to examine the effects of extreme temperatures on *Bulinus* species in water bodies and provide theoretical insights into biological control strategies for *Bulinus*, as well as explore the mechanisms by which molluscs adapt to extreme environments.

## 2. Results

### 2.1. Responses of B. globosus to Extreme Temperature Stress

**Figure 1:**
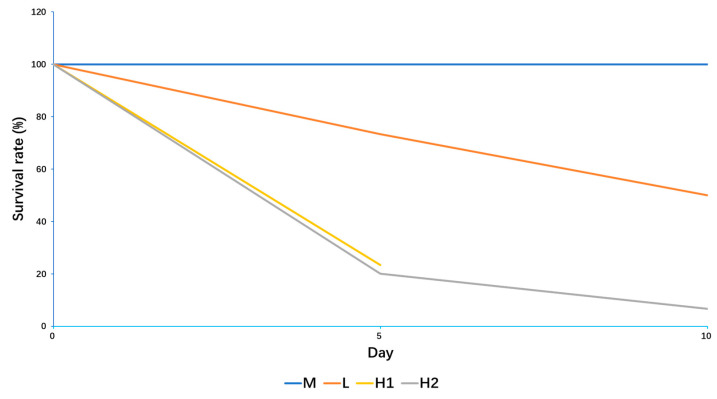
*B. globosus*’s survival curve under different temperature stress conditions.

The results indicated that the H group exhibited the lowest survival rate after 10 days of treatment, followed by the L and M groups. Notably, the H2 group exhibited the lowest survival rate within the H group, with rates also being lower than those of the M group. The survival rates for the H2, H1, L, and M groups were 6.67% (2/30), 23.33% (7/30), 50.00% (15/30), and 100% (30/30), respectively (Figure 1). In the chi-square test, the expected count of cells was more than five, and the sample size exceeded 40. The chi-square value from the Pearson chi-square test was 60.471, with a *p*-value of <0.0001, indicating that the differences among the data were statistically significant, suggesting variations in survival rates under different temperature conditions. After treatment, 54 snails were sequenced from the H, L, and M groups.

### 2.2. RNA Sequencing and De Novo Assembly

After removing adapter contamination, low-quality sequences, and unidentified bases, the valid data for the ten samples ranged from 5.44 to 7.56 Gb. The Q20 content in each sample exceeded 96%, while the Q30 content was greater than 93% (Table S1). These indicators demonstrate that the transcriptome sequencing data are of high quality and are suitable for subsequent analysis.

The clean data from all processed samples were subjected to de novo assembly, followed by normalization to obtain transcripts and Unigenes. The GC content of the Unigenes was 33.98%, with an average length of 1139.54 bp and a maximum length of 29,157 bp. The majority of Unigenes ranged from 300 to 900 bp in length, and the N50 value was 2042 bp (Figure S1 and Table S2). The Pearson correlation coefficient of the snail samples exposed to different temperature conditions revealed that the biological replicates at each temperature had a correlation coefficient greater than 0.99, indicating exceeding consistency among replicates and reliable data (Figure S2).

### 2.3. Unigene Annotation and Functional Classification

Through de novo transcriptome sequencing, a total of 93,686 Unigenes were successfully assembled and aligned with six major databases: GO (Gene Ontology), KEGG, NR (NCBI nonredundant protein sequences), eggNOG (evolutionary genealogy of genes: Non-supervised Orthologous Groups), Pfam, and Swiss-Prot. The annotation rates for these databases were 13.73%, 9.93%, 14.52%, 15.06%, 20.35%, and 26.81%, respectively (Table S3).

**Figure 2:**
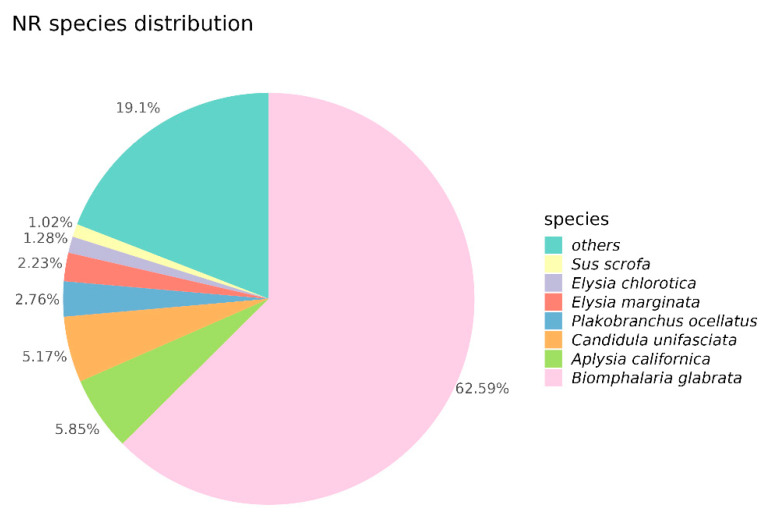
Results of the NR annotation for the Unigene of *B. globosus* under different temperature stress conditions.

**Figure 3:**
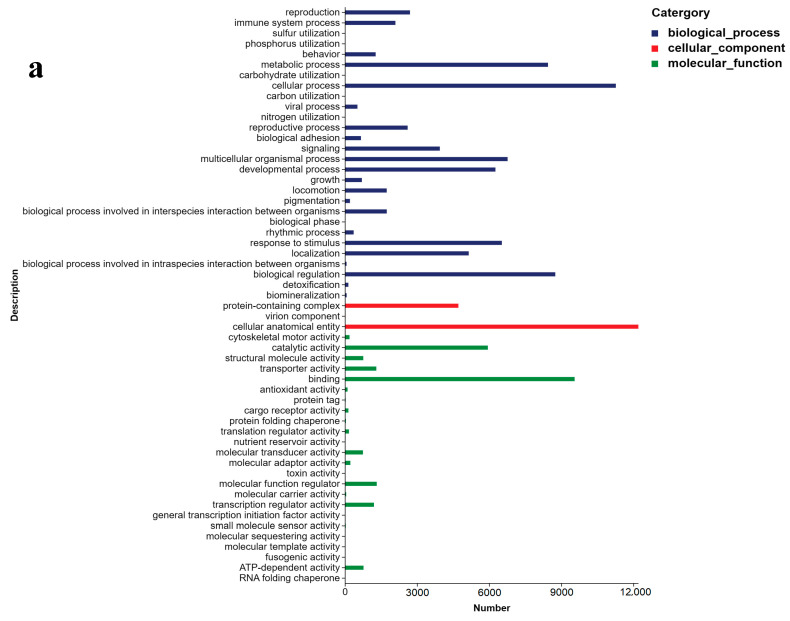

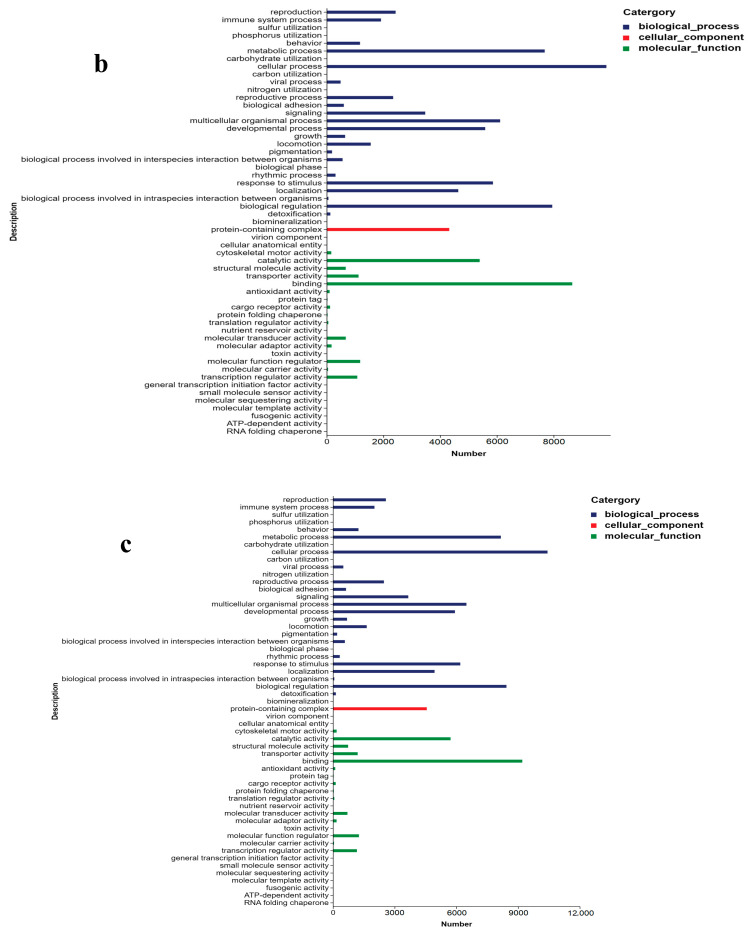
GO analysis for the Unigene of *B. globosus* under different temperature stress conditions: (**a**) all groups, (**b**) H group, (**c**) L group. Unigenes were categorized into three main GO domains: biological process (BP), cellular component (CC), and molecular function (MF).

**Figure 4:**
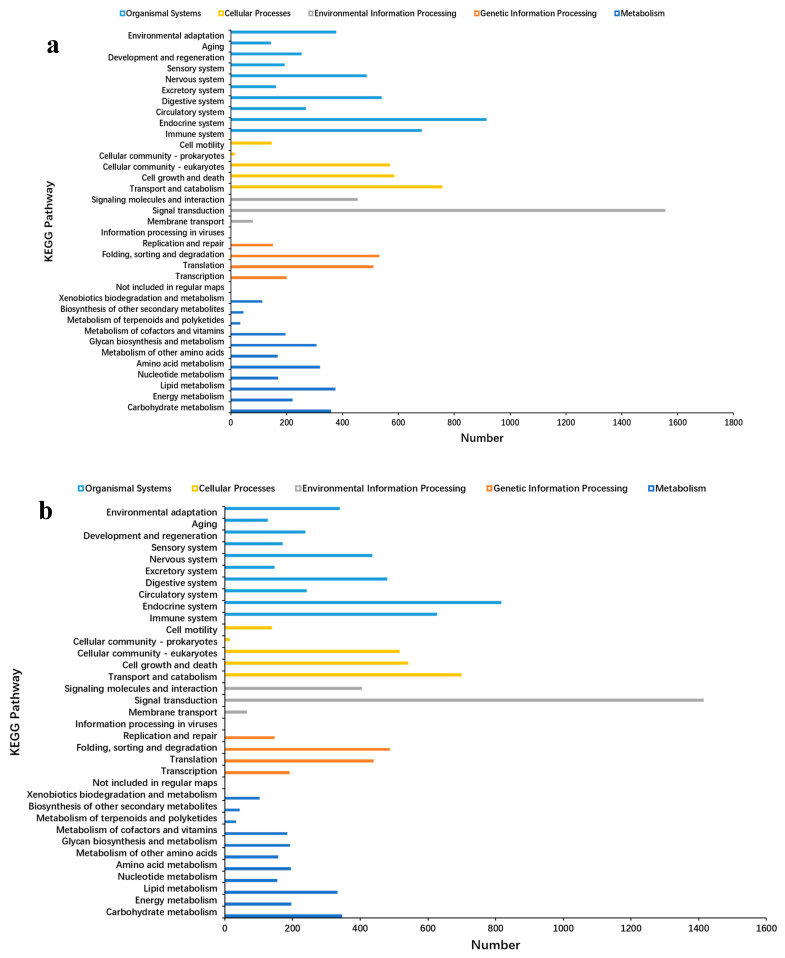

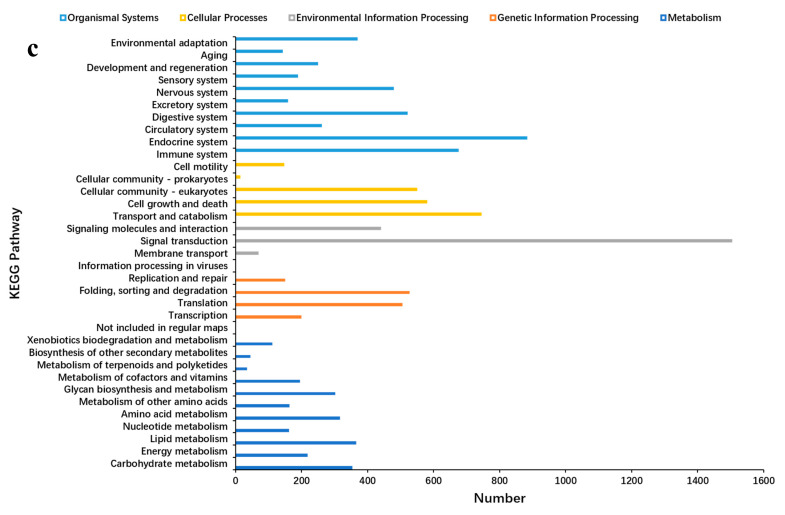
Analysis of the KEGG pathway for the Unigene of *B. globosus* under temperature stress: (**a**) all groups, (**b**) H group, (**c**) L group. The Unigene was mapped to five distinct KEGG subsystems, including organismal systems, genetic information processing, metabolism, cellular processes, and environmental information processing.

The results of the NR annotation revealed that 62.59% of the Unigenes were homologous to *B. glabrata*, 5.85% to *Aplysia californica*, 5.17% to *Candidula unifasciata*, and 2.76% to *Plakobranchus ocellatus* (Figure 2). This indicates a close evolutionary relationship between *B. globosus* and *B. glabrata*. Unigenes were categorized into three main GO domains: biological process (BP), cellular component (CC), and molecular function (MF). In terms of BP, the Unigenes were primarily associated with biological regulation, multicellular organism processes, responses to stimuli, and developmental processes. For CC, enrichment was observed in cellular anatomical entities and protein-containing complexes. Regarding MF, the Unigenes were predominantly annotated for molecular binding and catalytic activity (Figure 3a–c). The Unigenes were mapped to five distinct KEGG subsystems, including organismal systems, genetic information processing, metabolism, cellular processes, and environmental information processing. Among these, lipid metabolism was the most enriched, followed by carbohydrate metabolism, amino acid metabolism, energy metabolism, and glycan biosynthesis and metabolism (Figure 4a–c).

### 2.4. Analysis of DEGs Under Temperature Stress

**Figure 5:**
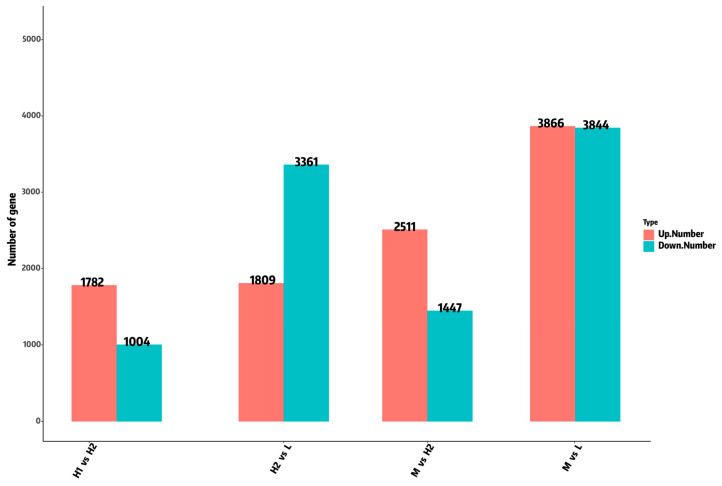
The number of genes differentially expressed by *B. globosus* under different temperature stress conditions.

Pairwise comparisons of gene expression profiles among experimental groups revealed substantial differences. Specifically, the L and M groups exhibited 3866 up-regulated genes and 3844 down-regulated genes. In comparison, the L and H2 groups showed even higher levels of up-regulation and down-regulation, with 1809 and 3361 genes affected, respectively. Furthermore, when comparing the M group with the H2 group, 2511 genes were up-regulated, while 1447 genes were down-regulated. Lastly, the H1 group demonstrated the up-regulation of 1782 genes and the down-regulation of 1004 genes compared to the H2 group (Figure 5). These results indicate that the gene expression of *B. globosus* was significantly altered under different temperature stress conditions.

**Figure 6:**
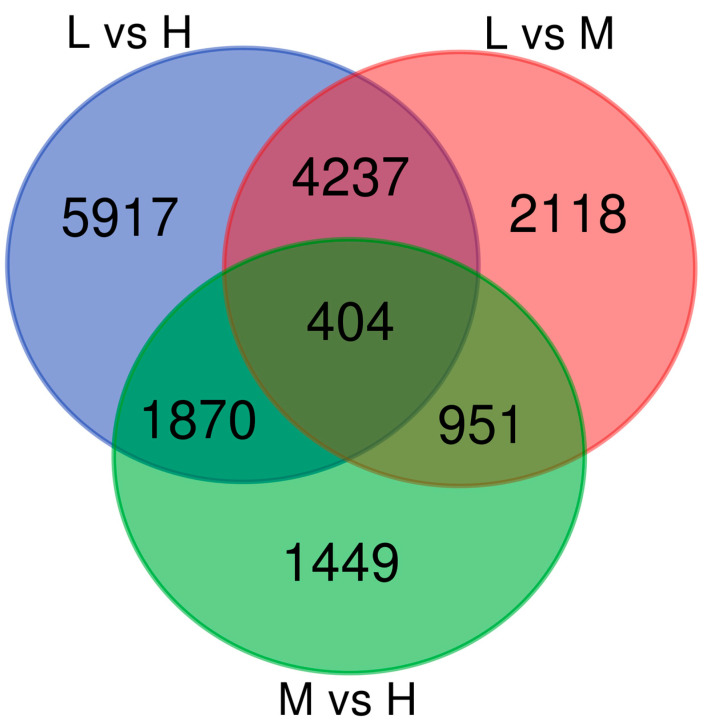
Venn analysis of the DEGs of *B. globosus* in different comparison groups.

Venn diagram analyses were conducted on the DEGs among various temperature comparison groups. Without accounting for the effect of experimental duration, 7710, 15,459, and 9348 DEGs were specifically expressed in the comparisons of the L vs. M group, the L vs. H group, and the M vs. H group, respectively, while 404 DEGs were commonly expressed across all three comparisons (Figure 6). Based on an analysis and review of the relevant literature, a preliminary selection of temperature-related genes was made from the DEGs uniquely identified in the three comparisons. After removing unmatched genes, 35 candidate genes related to temperature stress were identified (Table S4). These genes are potentially involved in the response to temperature stress in *B. globosus*.

### 2.5. GO and KEGG Analyses of DEGs

**Figure 7:**
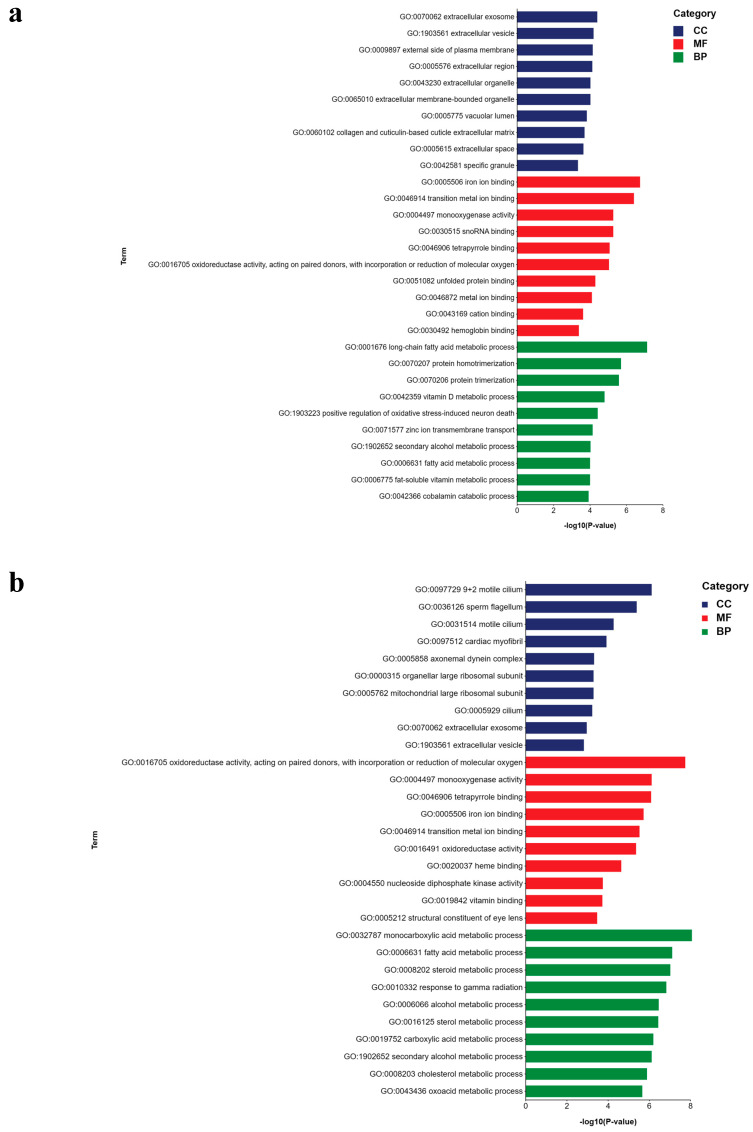

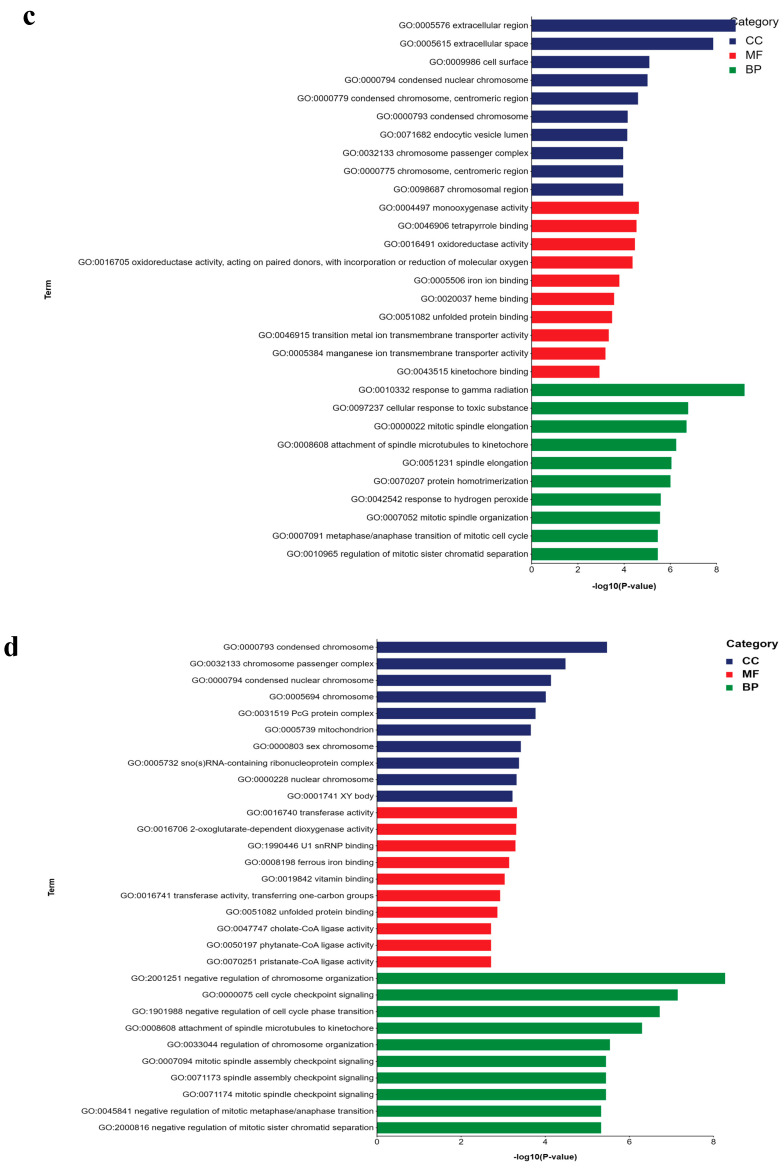
GO enrichment analysis of DEGs associated with different stress conditions in *B. globosus*: (**a**) L vs. M, (**b**) H vs. M, (**c**) H1 vs. H2, and (**d**) H vs. L. GO was classified according to molecular the function (MF), biological process (BP), and cellular component (CC). The top 10 GO term items with the smallest p-value, that is, the most significant enrichment, in each GO classification were selected for display.

**Figure 8:**
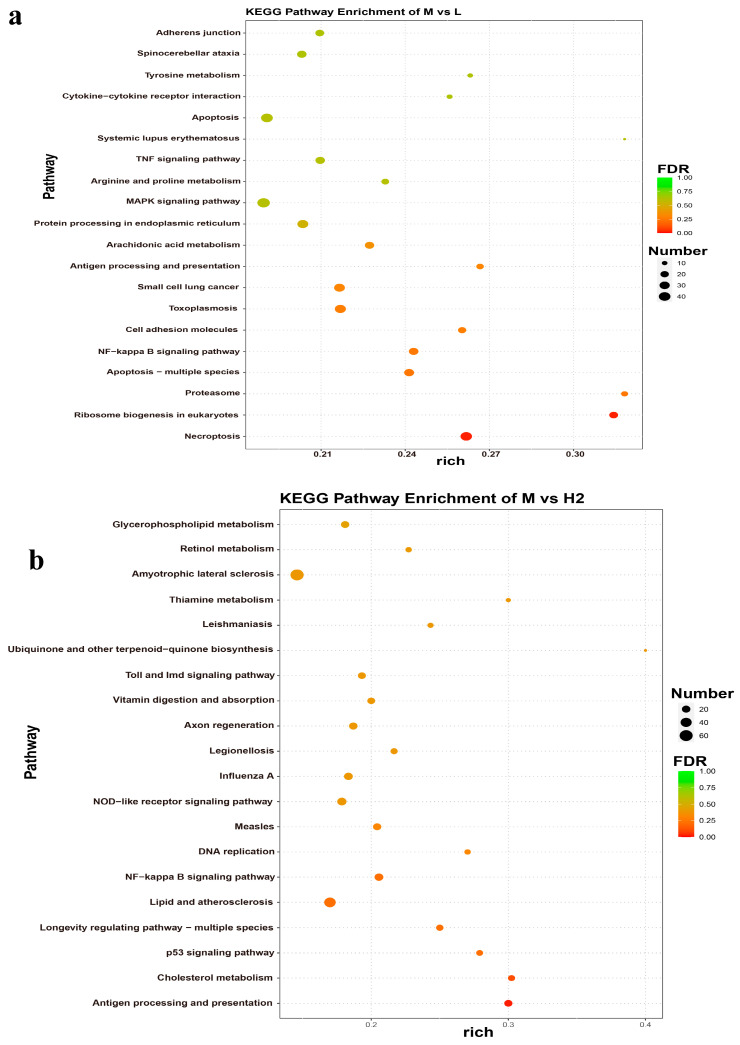

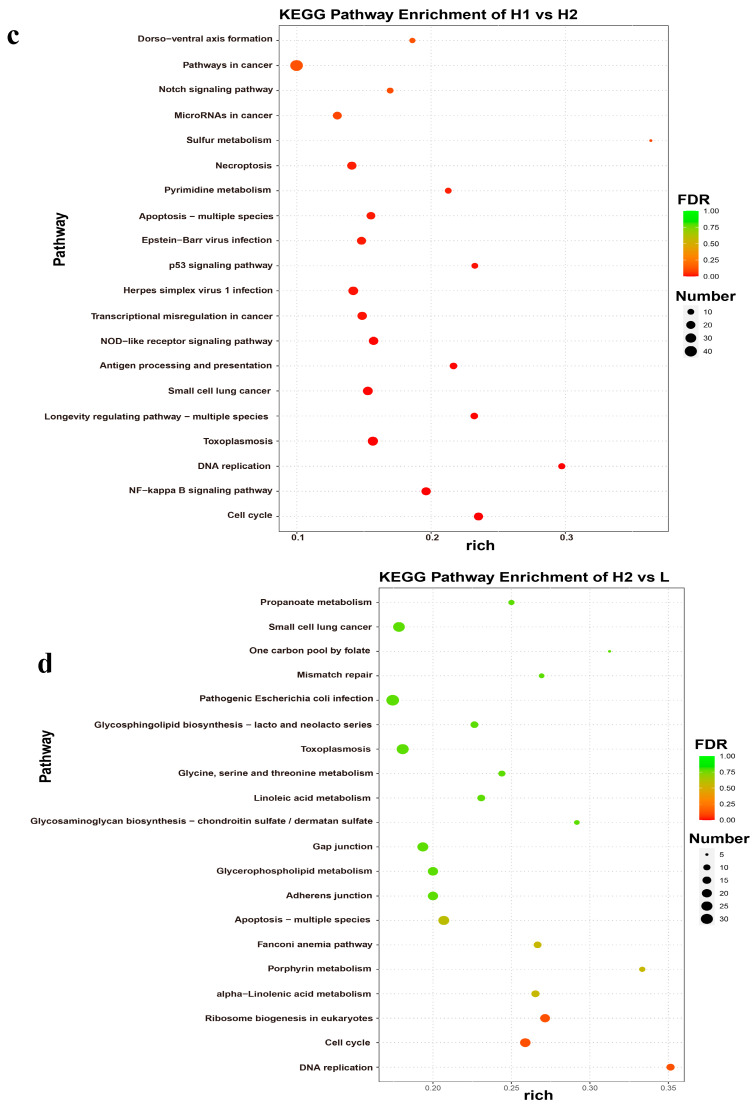
KEGG enrichment analysis of DEGs in *B. globosus*: (**a**) L vs. M, (**b**) H vs. M, (**c**) H1 vs. H2, and (**d**) H vs. L. The degree of enrichment was measured using the Rich factor, the FDR value, and the number of genes enriched on this pathway. Among them, the Rich factor refers to the ratio of the number of differentially expressed genes enriched in this pathway to the number of differentially expressed genes annotated. The larger the Rich factor, the greater the degree of enrichment. The general value range of the FDR is from 0 to 1. The closer it is to zero, the more significant the enrichment.

To identify genes with significant differences between the temperature stress groups and the control group, pairwise comparisons were conducted using the topGO software. The DEGs potentially associated with temperature stress in *B. globosus* were analyzed through GO enrichment. The top 10 GO terms with the smallest *p*-value in each category were selected for display, and the results are presented in Figure 7. Following cold stress (L vs. M), the DEGs were significantly enriched in 755 GO terms, including 551 terms related to BP, 79 to CC, and 125 to MF (Figure 7a). Among the BP terms, ‘long-chain fatty acid metabolic process’ emerged as the most significant, followed by ‘protein homotrimerization’. Several genes were also associated with notable categories, such as the ‘positive regulation of oxidative stress-induced neuron death’ and the ‘fatty acid metabolic process’, which may contribute to resistance and adaptation to cold in *B. globosus*. Within the MF category, ‘iron ion binding’ was highly represented. Furthermore, these 520 genes were classified into 21 KEGG functional pathways, demonstrating significant enrichment in the MAPK signaling pathway, apoptosis, and necroptosis (Figure 8a).

Under heat stress, DEGs were associated with 962 GO terms. The two most significantly annotated GO biological process pathways were ‘monocarboxylic acid metabolic process’ and ‘oxidoreductase activity, acting on paired donors, with the incorporation or reduction of molecular oxygen’ (Figure 7b). KEGG enrichment analysis showed that 524 DEGs were enriched in 30 KEGG pathways, including those associated with amyotrophic lateral sclerosis, lipid and atherosclerosis, and apoptosis. Notably, these DEGs were enriched in the amyotrophic lateral sclerosis pathway (Figure 8b).

DEGs were associated with 1109 GO terms in the comparison between the short-term and long-term heat stress groups (H1 vs. H2). The two primary significantly annotated BP pathways were the “response to gamma radiation” and the “extracellular region” (Figure 7c). KEGG analysis indicated that 524 DEGs were enriched in 39 KEGG pathways (Figure 8c).

DEGs were associated with 640 GO terms between the heat stress group and the cold stress group (H2 vs. L). The most significantly annotated BP pathway was the ‘negative regulation of chromosome organization’ (Figure 7d). KEGG analysis indicated that 330 DEGs were enriched in 20 KEGG pathways (Figure 8d).

### 2.6. Validation of RNA-Seq Results Using qRT-PCR

To validate the RNA-seq results, we selected nine DEGs related to temperature adaptation across various signaling pathways for qRT-PCR analysis. The results indicated that the expression trends of the genes in the different experimental temperature groups were basically consistent with the transcriptome sequencing results. These results suggest that the RNA-seq data are reliable and can be effectively utilized for bioinformatic analysis (Figure S3).

## 3. Discussion

The transmission of an infectious disease requires three essential factors: a source of infection, a transmission pathway, and a susceptible population. These factors are interdependent and work synergistically to influence the spread of infectious diseases. In the case of human schistosomiasis caused by *Schistosoma haematobium*, human patients serve as the source of infection, while *Bulinus* acts as the transmission pathway or vector. The human population is generally susceptible due to a lack of immunity [23]. Temperature is a critical driver of the distribution patterns of *B. globosus*. Over the past century, global warming and associated climate changes have intensified. The Fifth Assessment Report of the Intergovernmental Panel on Climate Change (IPCC), published in September 2013, forecasts that by the end of this century, the global average surface temperature is expected to rise by 1.5 to 4.8 °C, with extreme weather events becoming more frequent [24,25]. Global warming and the increase in extreme weather events will inevitably influence the distribution of *B. globosus*. Understanding the molecular mechanisms underlying the impact of temperature stress on *B. globosus* survival is crucial for providing a scientific basis for developing policies and measures to control schistosomiasis.

Currently, there is insufficient information regarding the gene expression profiles of *Bulinus* under temperature stress. This study used assembled de novo RNA-seq technology to investigate the transcriptomic changes in the tissues of *B. globosus* subjected to different temperature treatments, with the aim of preliminarily elucidating the molecular mechanisms underlying its response to temperature stress. Functional annotation and classification provided predictive insights into cellular metabolic pathways and condition-specific gene expression behaviors. In this study, 12,865 (13.73%) Unigenes were successfully annotated in the GO database. However, due to the lack of specific genomic information for the *Bulinus* genus, the majority of the Unigenes remained unannotated (86.27%). We attempted to align our data with the only available reference genome for *Bulinus truncatus*, but the alignment rate was a mere 4.95%. We then compared it with the reference genome of *Biomphalaria glabrata*, which is more closely related, but the matching rate was even lower at 0.26%. Given these low alignment rates, we had no choice but to proceed with a de novo transcriptome analysis. According to the GO classification, the cellular process and the cellular anatomical entity and binding constituted the largest functional groups in the three main GO categories: BP, CC, and MF, respectively. Similar results were observed by Habib in transcriptomic studies of *Bulinus truncatus* under *S. haematobium* infection [26]. KEGG pathway analysis revealed that lipid metabolism was the most annotated metabolic pathway. Similarly, a study on *Pomacea canaliculated* under cold stress found that the up-regulation of unsaturated fatty acid synthesis genes mitigated the adverse effects on membrane fluidity [27]. In summary, the functional annotation and classification of Unigenes provide a general profile of gene expression characteristics in *B. globosus*, contributing not only to further studies on this species but also offering insights for other species within the *Bulinus* genus.

Extreme temperatures often adversely affect the survival and biological functions of ectothermic animals [28]. To mitigate these negative effects, ectothermic species can adapt to temperature stress through physiological adjustments triggered by exposure to extreme temperatures [29]. Different types of temperature stress involve a wide range of molecular and physiological changes [30]. Liu and Xiao analyzed the cold resistance of *Pomacea canaliculata* using a transcriptomic approach, revealing the molecular mechanisms underlying cold resistance. This research provides a scientific basis for predicting and preventing the detrimental effects of *P. canaliculata* [27,31]. *Echinolittorina* snails exhibited elevated levels of heat shock protein (HSP)70 expression under high thermal stress. High levels of HSP70 mRNA can function as an adaptive mechanism to cope with locally stressful thermal environments [32]. In the case of the Mediterranean snail Xeropicta derbentina under heat stress, previous studies have demonstrated that efficient antioxidant defense mechanisms are activated following heat exposure, with varying temperature-dependent increases in activity. The activities of catalase and glutathione peroxidase, both antioxidant enzymes, exhibit distinct optimal responses to temperature, thereby complementing the Hsp70 response and each other when external temperature rise [33]. Based on RNA-seq results, DEGs related to lipid metabolism, carbohydrate metabolism, homeostasis regulation, and the antioxidant system in response to temperature stress were identified, and their most significant aspects were explored.

The alteration in temperature affects the permeability of biofilms, causing the cell membrane to transition from a liquid phase to a thin gel phase, thereby influencing membrane fluidity and the functional properties of membrane proteins and membrane-binding proteins [34,35]. Under cold conditions, plants, microorganisms, and animals increase the proportion of unsaturated fatty acids in the phospholipids that constitute cell membranes [36]. Following cold acclimation, genes involved in the synthesis of cholesterol, fatty acids, and unsaturated fatty acids exhibit significantly higher expression levels. Long-chain and very-long-chain fatty acids are essential components of cell membranes. Consequently, enhancing the expression of genes involved in the biosynthesis of long-chain and very-long-chain fatty acids in *B. globosus* alleviates the adverse effects of low temperatures on membrane fluidity. Compared to M, the DEGs of *TRINITY_DN1812_c3_g1* in the H and L groups were significantly enriched in the retrograde endocannabinoid signaling pathway, with expression levels up-regulated by 1.53 and 2.94 times, respectively. This increased expression of *N-acyl-phosphatidylethanolamine-hydrolysing phospholipase D* (*NAPE-PLD*) genes may enhance N-acylethanolamine biosynthesis. By hydrolyzing the phosphodiester bonds in N-acylphosphatidyl ethanolamine, NAPE-PLD genes can produce a broad spectrum of NAEs that function as lipid signaling molecules, playing a crucial role in regulating physiological processes under extreme temperature stress [37].

Chitin is a large structural polysaccharide composed of chains of modified glucose units. It is commonly found in the exoskeletons of insects and in the hard structures of invertebrates and fish. Chitin synthase, one of the key enzymes involved in chitin production, synthesizes this structural compound to form the exoskeleton and peritrophic membrane [38]. Chitinase, an enzyme associated with chitin digestion, breaks down the polymer into a soluble form that can be absorbed by the organism, facilitating the construction of a new exoskeleton [39]. Research indicates that the fall armyworm, *Spodoptera frugiperda*, tolerated high temperatures by regulating chitin-related genes [40]. In our study, the expression of chitinase and chitin synthase genes was found to be elevated in the H group. In snails exposed to elevated temperatures, chitinase (chitin-degrading enzyme) can still maintain its activity by hydrolyzing the β-1,4-glycosidic bonds in chitin to generate smaller sugar molecules, such as N-acetylglucosamine. These degradation products can further participate in the organism’s sugar metabolic pathways, serving as energy sources or precursors for the synthesis of other biological molecules. Furthermore, since chitin is the main component of fungal cell walls, chitinase may also indirectly participate in the regulation of sugar metabolism by hydrolyzing chitin in fungal cell walls to resist fungal infections at high temperatures. On the contrary, chitin synthase (chitin-synthesizing enzyme) may also participate in sugar metabolism at high temperatures, but it mainly catalyzes the synthesis of chitin. When the organism needs to synthesize new chitin structures or repair damaged chitin, chitin synthase plays a crucial role [41]. Next, we plan to verify the potential functions of chitin-related genes for the high-temperature tolerance of *B. globosus* via RNA interference or CRISPR/Cas9 technology.

Earlier studies have demonstrated that carbohydrate and lipid metabolism pathways serve as mechanisms for snails to obtain energy during winter periods. Propanoate metabolism plays a crucial role in low-temperature oxidation [42]. This metabolic pathway encompasses the biochemical processes involved in the synthesis, degradation, and utilization of propanoate, a three-carbon short-chain fatty acid. By producing substrates for the TCA cycle, propanoate metabolism facilitates ATP synthesis during cellular respiration [43]. *Acyl-CoA oxidase (ACOX)* activity increased to mobilize stored fats for energy under extreme temperatures and food in *Caenorhabditis elegans* [44]. In our study, an *ACOX* gene was observed to be up-regulated, and five genes (the *3-hydroxyisobutyryl-CoA hydrolase*, *4-aminobutyrate aminotransferase*, *succinyl-CoA synthetase alpha subunit*, *methylmalonyl-CoA decarboxylase*, and *acetyl-CoA synthetase* genes) were down-regulated in the snails of the L group. These findings indicated that *B. globosus* can enhance ATP from energy-supplying substrates by up-regulating the *ACOX* gene and down-regulating the five genes, thereby reducing energy metabolism efficiency and subsequently the cold resistance.

Previous studies have demonstrated that maintaining intracellular homeostasis can enhance the temperature resistance of snails [31]. Certain heat-related proteins provide protection against stress in these organisms [45]. HSP70, a molecular chaperone involved in protein folding, stabilizes protein structures and promotes correct folding, which is closely linked to the organism’s ability to withstand stress [46]. One study indicated that the HSP70 transcript level increases with temperature stimulation in both female and male golden apple snails [47]. In this paper, the DEGs of HSP70 showed up-regulation in both the H and L groups. The levels of Hsp70 increased significantly when the fish were subjected to acute temperature changes. The increased expression of Hsp70 may contribute to an increase in the stress response in this species [48]. High levels of inducible Hsp70 indicated high levels of protein damage, and the tolerance for further thermal stress increased in the fish [49,50]. However, Song et al. showed that HSP70 transcript levels in golden apple snails (*Pomacea canaliculata*) decreased slightly under cold shock and increased significantly under heat-shock conditions in both sexes compared to normal temperatures (26 °C) [47]. These results suggest that different poikilothermic animals may use a different strategy to handle temperature stress [51]. The responses of Hsp70 to more complex thermal signals and their ecological consequences in snails require further research, for which the present study may provide a foundational basis. The HSP-12.2-like proteins are small heat shock proteins (sHSPs), a subgroup of the HSP family characterized by low molecular weight (usually below 30 kDa). As a chaperone protein, it assists in the proper folding of proteins and plays a key role in degrading misfolded proteins [52]. In our experiment, the DEGs of the Hsp-12.2-like protein were up-regulated in *B. globosus* under temperature stress. Cold shock proteins (CSPs) help maintain RNA stability and assist in proper protein folding, thereby minimizing the physiological disruption caused by cold environments [53]. The DEGs of cold shock proteins (CSPs) were not detected in this paper. Experimental findings reveal that *B. globosus* exhibit poor tolerance to low temperatures, which may also be related to the absence of CSP expression.

A significant increase in reactive oxygen species is observed during temperature stress [54]. To address the challenges posed by temperature stress, antioxidant factors such as superoxide dismutase (SOD) and glutathione peroxidase (GPX) exhibit increased activity [55]. GPX is an antioxidant enzyme that scavenges reactive oxygen species, and *C. gigas* enhances its antioxidant capacity by up-regulating GPX [56]. This enzyme plays a critical role in protecting cells from oxidative stress. In this study, one DEG of GPX was observed to be down-regulated in the low-temperature group and up-regulated in the high-temperature group. The glutathione peroxidase-like isoform X2 (GPX-like X2) refers to a variant of the glutathione peroxidase enzyme family that shares structural or functional similarities to known glutathione peroxidases. To protect against oxidative stress caused by temperature fluctuations, organisms rely on antioxidant enzymes that effectively scavenge reactive oxygen species [57]. High temperatures or environmental exposure can increase oxidative stress in cells, leading to the up-regulation of GPX-like X2. Conversely, reduced temperatures can decrease metabolic activity, reducing the expression or activity of GPX-like X2.

There are several limitations in this study, although valuable information provided insights into the molecular responses of *B. globosus* under extreme temperature stress. First, this study considered treatment time in the high-temperature group, as treatment time is also an influencing factor leading to the death of snails. The H2 group was included with the aim of identifying time-related differential genes. However, we did not establish subgroups based on treatment time in the L and M groups, resulting in a small sample size that may introduce errors and confound the effects of time and temperature. Second, the limitations of de novo transcriptome assembly stem from technical, computational, and biological challenges that existed particularly in non-model organisms. However, this study provides the first transcriptomic data on the effect of temperature stress duration, enhancing genomic resources for annotation and discovery and offering new insights into critical genes for rising temperatures under climate change. These findings not only improve our understanding of the impacts of temperature stress on *Bulinus* and its global distribution but also establish a foundation for future research into targeted control strategies. Further studies could explore the functional roles of these genes and their potential as intervention points for schistosomiasis. This study provides a theoretical framework for other poikilotherm hosts under temperature stress.

## 4. Materials and Methods

### 4.1. B. globosus Preparation

Laboratory-bred *B. globosus* snails propagated on Pemba Island, Zanzibar, were selected for experimentation. Adult snails, aged 10 weeks and from the same generation, served as the experimental subjects. The snails were randomly assigned to beakers and were subjected to different temperature treatments, which were coded accordingly: the low-temperature group (L group, 10 °C), the normal-temperature group (M group, 26 °C), and the high-temperature group (H group, 35 °C). Each group comprised 10 adult snails of similar age and was reared in climate incubators at their respective temperatures for 10 days. The snails were maintained under a controlled photoperiod of 12 h of light and 12 h of darkness, with an illumination intensity of 1000 lx, which was provided by a general environmental experiment box. They were kept in 500 mL beakers filled with dechlorinated tap water. The L and M groups included three parallel subgroups, while the H group contained six parallel subgroups. Specifically, the H1 subgroup consisted of three parallel groups maintained at 35 °C for 5 days, and the H2 subgroup consisted of three parallel groups maintained at 35 °C for 10 days. (Only one parallel subgroup had surviving snails after 10 days.) The surviving snails from each group were collected as samples for de novo transcriptome sequencing. This study was approved by the ethics review departments of our Zanzibar partners (ZAHREC/01/PR/JUNE/2024/21) and the Jiangsu Institute of Parasitic Diseases (JIPD-2023-012), and it also adhered to the principles of experimental animal management practices.

### 4.2. RNA Sequencing

After treatment with temperature, living snails were collected and dissected. Whole soft tissues from snails were isolated and immediately frozen at −80 °C to maintain the integrity of the RNA. The RNA 6000 Nano Kit (Agilent, Santa Clara, CA, USA) was utilized to extract total RNA from each sample, with separate gene pools prepared for each sample. To assess the quality and quantity of the RNA, a Nanodrop spectrophotometer, agarose electrophoresis, and the Agilent 2100 bioanalyzer method were used to measure RNA purity, concentration, and structural integrity. Total RNA was treated with two rounds of oligo(dT)-tagged magnetic bead selection to isolate poly(A)-tagged RNA. Isolated RNA was reverse transcribed under high-temperature and divalent cation conditions to generate cDNA libraries with an average insert size of 300 bp (±50 bp) for paired-end libraries. Library quality was evaluated using the Agilent 2100 bioanalyzer (Agilent, CA, USA) with the Agilent High Sensitivity DNA Kit (Agilent, CA, USA). qRT-PCR was performed to accurately quantify library concentration, ensuring quality control. The cDNA libraries were then loaded onto the Illumina HiSeq 4000 sequencing platform for high-throughput sequencing, generating reads of 150 bp.

### 4.3. De Novo Transcriptome Data Processing and Differentially Expressed Gene Analysis

Due to the limited molecular-level studies on *B. globosus*, de novo transcriptome analysis was used in this study [20]. Sequencing data were processed using a de novo assembly approach. Low-quality reads, such as adaptors, reads shorter than 50 base pairs, and reads with an average quality score below Q20, were removed using CutAdapt and Perl scripts [58]. Sequence quality was verified using FASTQC, and subsequent analyses were performed on high-quality clean data. De novo transcriptome assembly was carried out with Trinity (v2.15.1) [59]. The longest transcript within each cluster was selected as the Unigene. The gene function for the Unigene was annotated using the DIAMOND software (v. 5.1.0.49) [60]. The database used for gene function annotation included GO, KEGG, NR, eggNOG, Pfam, and Swiss-Prot. The DEG analysis was carried out using DESeq (v1.38.3) to compare expression differences between the two groups. DESeq was used to identify DEGs according to the following criteria: |log2FoldChange| > 1 and a significance threshold of the *P*-adj < 0.05 (adjusted *p*-values for multiple testing in the DEG analysis).

### 4.4. Annotation of the GO Function and Analysis of the KEGG Pathway

GO analysis of DEGs was performed using topGO (v2.50.0). The hypergeometric distribution method was used to calculate *p*, with a significance threshold of *p* < 0.05 used to identify significantly enriched GO terms. This analysis provides the primary biological functions of the DEGs. KEGG pathway analysis was carried out using clusterProfiler (v4.6.0), with special attention being given to pathways that were significantly enriched at *p* < 0.05.

### 4.5. qRT-PCR Analysis

A qRT-PCR experiment was conducted to validate the consistency of the RNA-seq analysis. Nine DEGs were selected based on their potential functional significance, and primers were designed using Primer-BLAST (Table 1). The quality and purity of the total RNA from the 10 sample groups were assessed using Nanodrop and agarose gel electrophoresis. Reverse transcription was performed using the PrimeScriptTM 1st Strand cDNA Synthesis Kit (Takara Bio, Shiga, Japan) to synthesize cDNA. The reference gene for the *Actin* gene of *Biomphalaria glabrata* was used as the control [61]. Amplification specificity was confirmed via melting curve analysis. The relative expression levels of the target gene were calculated using the comparative cycle threshold (Ct) method (2^−ΔΔCt^) [62]. Each qRT-PCR experiment included three biological and technical replicates for each sample.

### 4.6. Statistical Analysis

A Chi-square test was used to process and evaluate the data. When the expected count *T* of the chi-square analysis is ≥5 and the total sample size *n* is ≥40, the Pearson chi-square test results are used. All analytical functions were analyzed using the statistical software SPSS 20.0 (International Business Machines Corporation, Armonk, NY, USA). *p* < 0.05 was considered statistically significant.

## 5. Conclusions

Our results demonstrate that temperature plays a crucial role in regulating *Bulinus* spp., and the treatment duration significantly influences the survival rate of these snails. De novo transcriptome sequencing has revealed key genes involved in lipid metabolism, carbohydrate metabolism, homeostasis regulation, and the antioxidant system. KEGG pathway analysis identified significant enrichment of DEGs in heat shock protein pathways, propanoate metabolism, and N-acylethanolamine metabolism pathways. These results elucidate the molecular mechanisms driving the survival patterns of *B. globosus* in response to temperature stress at the transcriptional level. In summary, this study provides transcriptomic data on the effects of stress for the first time, thereby extending genomic resources for annotation and gene discovery while offering new insights into critical genes associated with extreme temperatures under climate change. These findings not only enhance our understanding of the impact of temperature stress on *Bulinus* and its global distribution but also establish a solid foundation for future research aimed at developing targeted schistosomiasis control strategies.

## Figures and Tables

**Table 1:** Primer sequences of differentially expressed genes of *B. globosus* under extreme temperature stress.

Gene ID	Forward Primer	Reverse Primer
TRINITY_DN14794_c0_g1	CGGTAGTAGTAGTTGAAGTAGCA	GTTATGGAGAATCTGAGGTAGGAA
TRINITY_DN6699_c1_g1	ACACCACCAGATTCATAA	TAACAGCCACATTACTTG
TRINITY_DN1701_c0_g2	GTACGATTGGCTGTTGTC	GGCTATGTGTGTTCTTTCTG
TRINITY_DN1300_c6_g1	TGTTCTGTTGTCCTCCTT	TCTGTTGACCACTCTGTT
TRINITY_DN14794_c0_g2	TGTATCCAGTGTCATAAGC	ATTGTTCGTCAACCAGTT
TRINITY_DN13712_c0_g1	CTTCCTCGCAATCACCTT	TTACTACAACTACAGCCAAGAA
TRINITY_DN4554_c0_g1	AGAGACTACTGTGATACTAC	ATGGCGTAATATCTGGAT
TRINITY_DN66651_c0_g1	TAACCTTGAATGACCAGAT	TGATACTAGCCACTATGC
TRINITY_DN4880_c0_g1	TACGAGTGGTGCTATATTAC	CAATCACGCAATGTATTCA
Actin	AATGAGCGATTCAGATGT	GATGGAGTTGTAGGTTGT

## Data Availability

The data presented in this study are available upon request from the corresponding author.

## Supplementary Materials

The following supporting information can be downloaded at https://www.mdpi.com/article/10.3390/ijms26115326/s1.
