# Supplementary material for: De Novo Transcriptome Assembly and Annotation Elucidate the Response to Extreme Temperature Stress in the Intermediate Host Bulinus globosus of Schistosoma haematobium
Source: Int J Mol Sci. 2025 Jun 1;26(11):5326. doi: 10.3390/ijms26115326 (PMC12154099; doi:10.3390/ijms26115326)
Supplement: Supplementary file 1 [file ijms-26-05326-s001.zip › ijms-3614767-supplementary.docx]

**Table S1.** Overview of sequencing quality control for *B. globosus* under different temperature stress


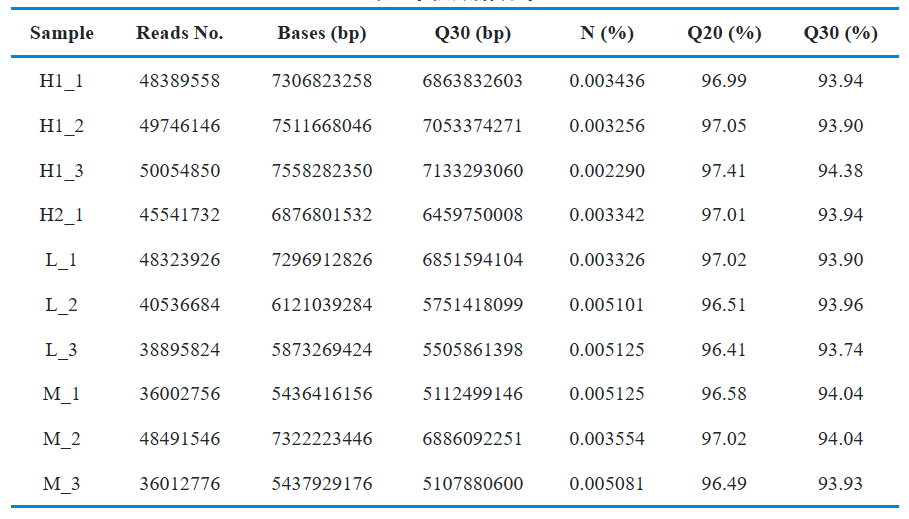


Note: Reads No.: Total number of reads obtained after sequencing; Bases (bp): Total number of base pairs in the sequencing data; Q30 (bp): Total number of bases with a quality score ensuring an accuracy rate of over 99.9%; N (%): Percentage of ambiguous (uncertain) bases in the sequencing data. Arabic numerals indicate biological repetition.

**Table S2.** Assembly results of the transcriptome data of *B. globosus* under different temperature stress


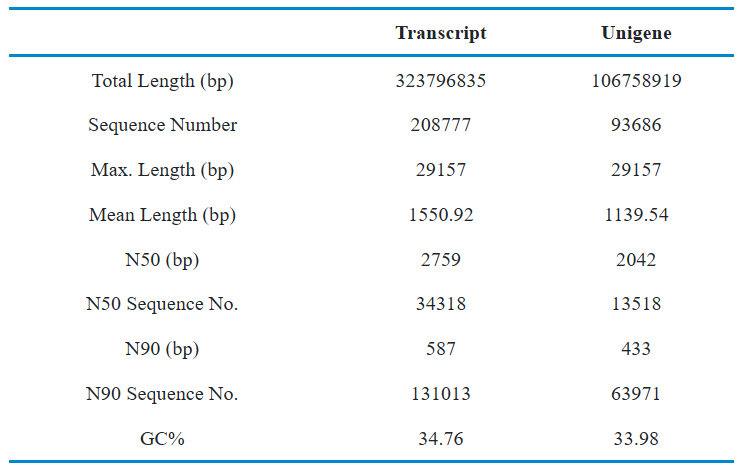


Note: Total Length (bp): total length of the sequence; Sequence Number: total number of sequences; Max. Length (bp): indicates the maximum length of the sequence; Mean Length: the average length of the sequence; N50 (bp): Arrange all sequences from long to short, add the length of the sequence in that order, and when the length of the addition reaches 50% of the total length of the sequence, the length of the last sequence; N90 (bp): All sequences are arranged by length from longest to shortest, and the length of the sequence is added in this order. When the length of the addition reaches 90% of the total length of the sequence, the length of the last sequence; N50 Sequence No.: The total number of sequences whose length is greater than N50; N90 Sequence No.: The total number of sequences longer than N90; GC%: The GC content of the sequence.

**Table** **S3.** Statistics of DIAMOND annotation results for Unigene of *B. globosus* under different temperature stress


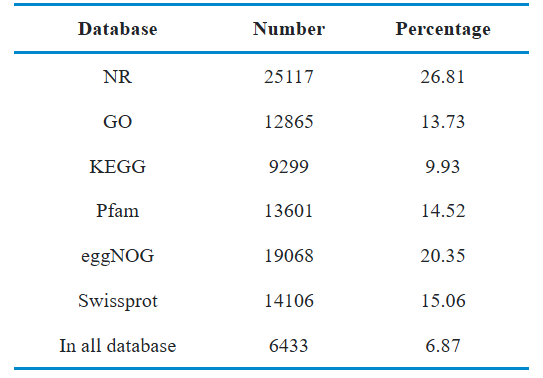


**Table S4.** Expression profiles of key genes of *B. globosus* under different temperature stress conditions

| **Gene_ID** | **Name** | **Description** | **H vs L log2fc** | **M vs H log2fc** | **M vs L log2fc** |
| --- | --- | --- | --- | --- | --- |
| TRINITY_DN31343_c0_g2 | KAF6392558.1 | hypothetical protein mPipKuh1_007759 [Pipistrellus kuhlii] | -5.85 | 2.39 | -3.45 |
| TRINITY_DN1525_c0_g1 | XP_013068346.1 | maternal embryonic leucine zipper kinase-like [Biomphalaria glabrata] | 3.44 | -2.19 | 1.27 |
| TRINITY_DN32848_c0_g1 | QNL15855.1 | polymorphic transmembrane cluster 2 transmembrane protein 2, partial [Biomphalaria glabrata] | -3.92 | 1.52 | -2.38 |
| TRINITY_DN1812_c3_g1 | XP_013078888.1 | N-acyl-phosphatidylethanolamine-hydrolyzing phospholipase D-like [Biomphalaria glabrata] | 1.39 | 1.53 | 2.94 |
| TRINITY_DN4835_c0_g1 | XP_013090869.1 | beta-1,3-galactosyltransferase 1-like [Biomphalaria glabrata] | -4.60 | 2.34 | -2.24 |
| TRINITY_DN19884_c0_g1 | XP_022810619.1 | neuromacin-like protein [Stylophora pistillata] | -3.00 | 6.35 | 3.36 |
| TRINITY_DN13712_c0_g1 | XP_013066615.1 | uncharacterized protein LOC106055044 [Biomphalaria glabrata] | 1.97 | -3.01 | -1.03 |
| TRINITY_DN30220_c0_g1 | CAG5123208.1 | unnamed protein product [Candidula unifasciata] | -1.05 | 2.10 | 1.06 |
| TRINITY_DN3920_c2_g2 | XP_013072202.1 | uncharacterized protein LOC106059183 [Biomphalaria glabrata] | -2.07 | -1.98 | -4.04 |
| TRINITY_DN26831_c0_g1 | AWP10000.1 | putative rhamnose-binding lectin-like isoform 2 [Scophthalmus maximus] | 1.22 | 1.44 | 2.68 |
| TRINITY_DN3644_c0_g2 | XP_013080921.1 | uncharacterized protein LOC106066446 [Biomphalaria glabrata] | -1.42 | 2.55 | 1.15 |
| TRINITY_DN17612_c1_g2 | XP_013081549.1 | uncharacterized protein LOC106067000 isoform X1 [Biomphalaria glabrata] | -3.33 | -2.50 | -5.82 |
| TRINITY_DN66651_c0_g1 | XP_013073788.1 | beta-1,3-galactosyltransferase 1-like [Biomphalaria glabrata] | -1.39 | -1.52 | -2.90 |
| TRINITY_DN1656_c0_g2 | XP_035828124.1 | cubilin, partial [Aplysia californica] | 3.19 | -4.40 | -1.20 |
| TRINITY_DN10822_c0_g1 | XP_013064352.1 | putative protein PHLOEM PROTEIN 2-LIKE A3, partial [Biomphalaria glabrata] | -3.16 | -1.85 | -5.00 |
| TRINITY_DN4880_c0_g1 | XP_013067328.1 | protein krueppel-like [Biomphalaria glabrata] | -2.89 | 1.48 | -1.40 |
| TRINITY_DN1300_c6_g1 | XP_013085878.1 | glutathione peroxidase-like isoform X2 [Biomphalaria glabrata] | -2.63 | 1.45 | -1.17 |
| TRINITY_DN1018_c1_g1 | XP_012944992.1 | protein quiver [Aplysia californica] | -2.45 | 1.04 | -1.39 |
| TRINITY_DN14794_c0_g2 | XP_013076884.1 | heat shock protein Hsp-12.2-like [Biomphalaria glabrata] | -3.16 | 5.25 | 2.11 |
| TRINITY_DN13825_c0_g1 | XP_013096136.1 | homeobox protein 3-like [Biomphalaria glabrata] | 3.00 | 1.35 | 4.36 |
| TRINITY_DN1701_c0_g2 | XP_012935630.1 | uncharacterized protein LOC101850152 [Aplysia californica] | -3.70 | 1.85 | -1.83 |
| TRINITY_DN54815_c0_g2 | AKC57283.1 | polyprotein, partial [Bemisia tabaci] | 2.91 | 2.62 | 5.56 |
| TRINITY_DN12905_c0_g1 | CAG5127535.1 | unnamed protein product [Candidula unifasciata] | -3.89 | 1.52 | -2.36 |
| TRINITY_DN3939_c0_g1 | XP_013072205.1 | uncharacterized protein LOC106059187 [Biomphalaria glabrata] | -1.18 | -2.25 | -3.41 |
| TRINITY_DN4554_c0_g1 | XP_013078718.1 | uncharacterized protein LOC106064659 [Biomphalaria glabrata] | -2.80 | 1.32 | -1.48 |
| TRINITY_DN3623_c1_g1 | CAG5117080.1 | unnamed protein product [Candidula unifasciata] | -4.50 | 2.12 | -2.36 |
| TRINITY_DN6699_c1_g1 | XP_013069954.1 | heat shock protein Hsp-12.2-like [Biomphalaria glabrata] | -4.98 | 6.70 | 1.74 |
| TRINITY_DN13041_c0_g1 | XP_013075489.1 | uncharacterized protein LOC106061821, partial [Biomphalaria glabrata] | -8.66 | 4.02 | -4.64 |
| TRINITY_DN2160_c0_g1 | XP_013090578.1 | beta-1,3-glucan-binding protein-like [Biomphalaria glabrata] | -1.16 | 4.03 | 2.89 |
| TRINITY_DN9635_c0_g1 | XP_013075837.1 | GTPase IMAP family member 7-like [Biomphalaria glabrata] | -4.15 | 1.58 | -2.55 |
| TRINITY_DN2315_c3_g1 | XP_013083783.1 | uncharacterized protein LOC106068828 [Biomphalaria glabrata] | -2.80 | 1.26 | -1.52 |
| TRINITY_DN14794_c0_g1 | XP_013069954.1 | heat shock protein Hsp-12.2-like [Biomphalaria glabrata] | -3.77 | 6.06 | 2.31 |
| TRINITY_DN264_c1_g1 | XP_013072073.1 | receptor-type tyrosine-protein phosphatase T-like isoform X2 [Biomphalaria glabrata] | -2.38 | 1.35 | -1.00 |
| TRINITY_DN715_c0_g1 | ABW86957.1 | histone 2B [Aplysia californica] | 1.29 | 3.21 | 4.51 |
| TRINITY_DN8822_c2_g1 | KAF2350396.1 | protein of unknown function DUF4371, partial [Trinorchestia longiramus] | 3.93 | -2.86 | 1.09 |


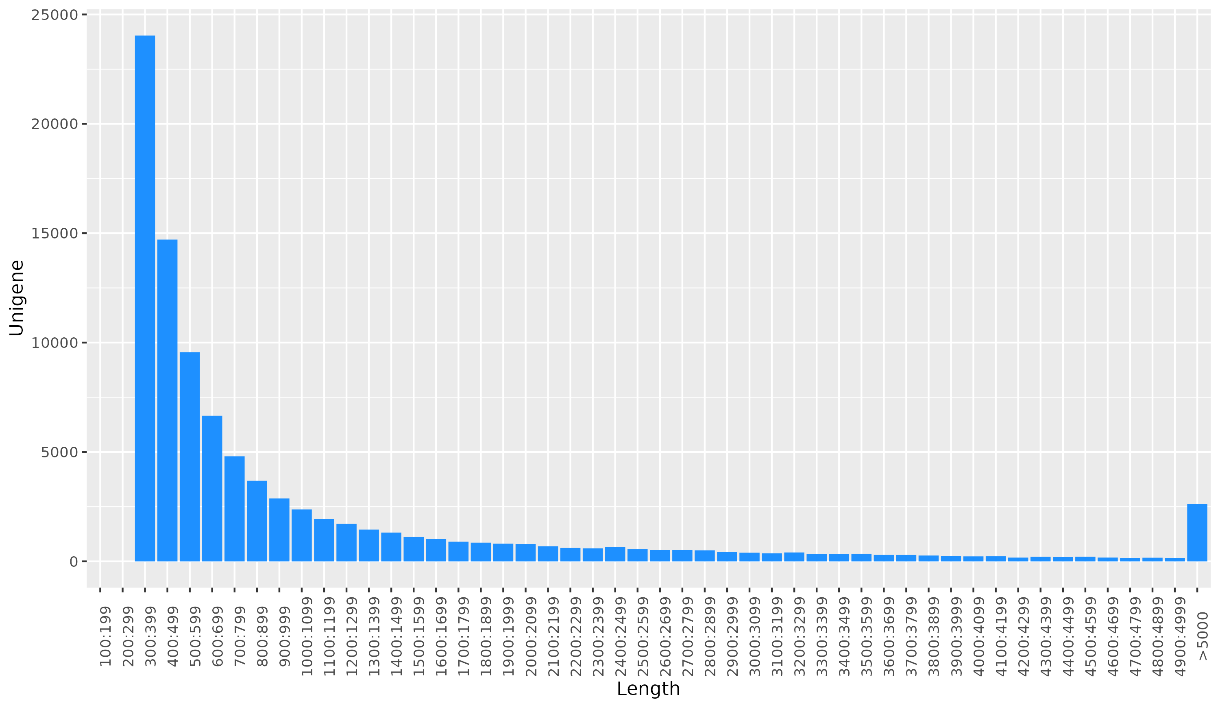


**Figure S1.** Length distribution of Unigenes in *B. globosus* subjected to different temperature stress


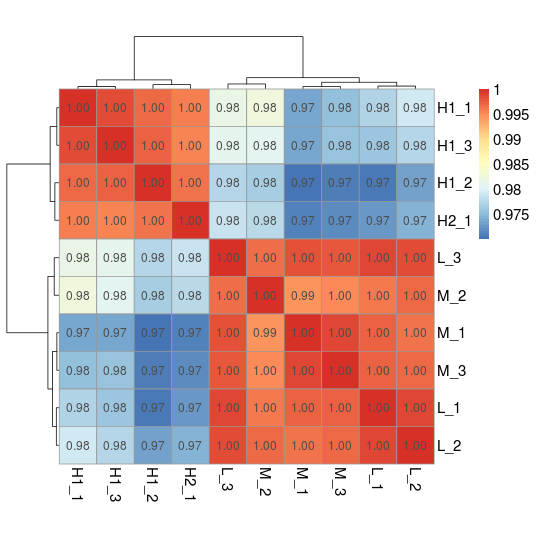


**Figure S2.** Heatmap of Pearson correlation coefficients among *B. globosus* under different temperature stress


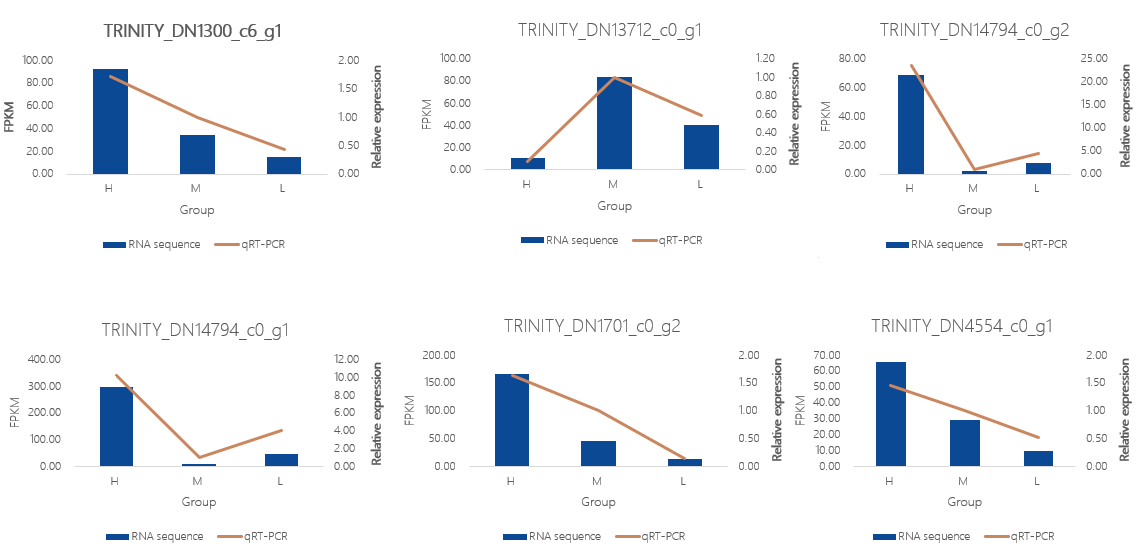


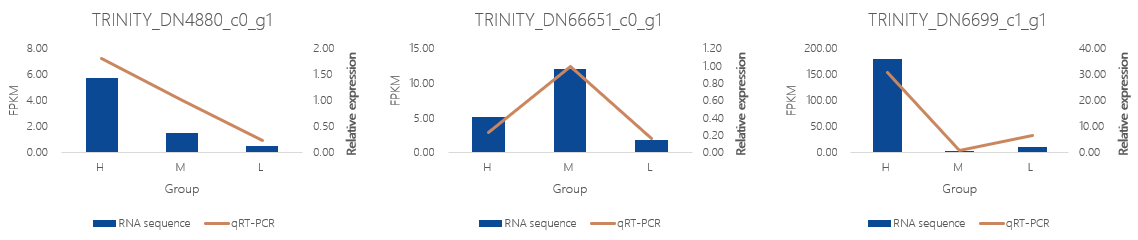


**Figure S3.** qRT-PCR validation of the relative expression levels of differentially expressed genes in *B. globosus* under temperature stress
